# Role of Serine140 in the mode of action of *Mycobacterium tuberculosis β*-ketoacyl-ACP Reductase (MabA)

**DOI:** 10.1186/1756-0500-5-526

**Published:** 2012-09-25

**Authors:** Leonardo A Rosado, Rafael Andrade Caceres, Walter Filgueira de Azevedo, Luiz A Basso, Diógenes S Santos

**Affiliations:** 1 Centro de Pesquisas em Biologia Molecular e Funcional (CPBMF), Instituto Nacional de Ciência e Tecnologia em Tuberculose (INCT-TB), Pontifícia Universidade Católica do Rio Grande do Sul (PUCRS), Av. Ipiranga 6681, Porto Alegre, RS, 90619-900, Brazil; 2 Programa de Pós-Graduação em Medicina e Ciências da Saúde, PUCRS, Av. Ipiranga 6681, Porto Alegre, RS, 90619-900, Brazil; 3 Faculdade de Biociências, Laboratório de Bioquímica Estrutural, PUCRS, Av. Ipiranga 6681, Porto Alegre, RS, 90619-900, Brazil; 4 Programa de Pós-Graduação em Biologia Celular e Molecular, PUCRS, Av. Ipiranga 6681 – Tecnopuc – Prédio 92A, ZIP CODE 90619-900, Porto Alegre, RS, Brazil

**Keywords:** *Mycobacterium tuberculosis*, β-Ketoacyl-ACP Reductase, MabA, Site-directed mutagenesis, Enzyme activity, Fluorescence spectroscopy, Molecular modeling

## Abstract

**Background:**

Tuberculosis (TB) still remains one of the most deadly infectious diseases in the world. *Mycobacterium tuberculosis* β-ketoacyl-ACP Reductase (MabA) is a member of the fatty acid elongation system type II, providing precursors of mycolic acids that are essential to the bacterial cell growth and survival. MabA has been shown to be essential for *M. tuberculosis* survival and to play a role in intracellular signal transduction of bacilli.

**Findings:**

Here we describe site-directed mutagenesis, recombinant protein expression and purification, steady-state kinetics, fluorescence spectroscopy, and molecular modeling for S140T and S140A mutant MabA enzymes. No enzyme activity could be detected for S140T and S140A. Although the S140T protein showed impaired NADPH binding, the S140A mutant could bind to NADPH. Computational predictions for NADPH binding affinity to WT, S140T and S140A MabA proteins were consistent with fluorescence spectroscopy data.

**Conclusions:**

The results suggest that the main role of the S140 side chain of MabA is in catalysis. The S140 side chain appears to also play an indirect role in NADPH binding. Interestingly, NADPH titrations curves shifted from sigmoidal for WT to hyperbolic for S140A, suggesting that the S140 residue may play a role in displacing the pre-existing equilibrium between two forms of MabA in solution. The results here reported provide a better understanding of the mode of action of MabA that should be useful to guide the rational (function-based) design of inhibitors of MabA enzyme activity which, hopefully, could be used as lead compounds with anti-TB action.

## Findings

### Background

Tuberculosis (TB) remains the leading cause of mortality due to a bacterial pathogen, *Mycobacterium tuberculosis*[1]. In 2008, there were an estimated 8.9-9.9 million incident cases of TB, killing two million people annually [2]. In addition, there were an estimated 0.5 million cases of multi-drug resistant TB (MDR-TB), which is defined as strains resistant to, at least, isoniazid and rifampicin [2]. The emergence of extensively drug-resistant (XDR-TB) TB cases, defined as cases in persons with TB whose isolates are MDR that are also resistant to a fluoroquinolone and, at least, one second-line injectable agent (amikacin, kanamycin and/or capreomycin) [2,3], its widespread distribution [4], and unprecedented fatality rate [5], raise the prospect of virtually incurable and deadly TB worldwide. Recently, a total new strain was identified as resistant to all first and second line of anti-TB drugs tested [6,7]. There is thus is an urgent need for the development of new antimycobacterial agents.

The type II fatty acid biosynthesis system (FAS-II) is present in bacteria, plants and organisms of the phylum Apicomplexa, but generally considered to be absent from mammals. *M. tuberculosis mabA(fabG1)*–encoded β-ketoacyl-ACP Reductase (MabA) is a member of FAS-II system, which elongates acyl fatty acid precursors yielding the long carbon chain of the meromycolate branch of mycolic acids, the hallmark of mycobacteria [8]. It has been shown that MabA is essential for *M. tuberculosis* survival [9]. Post-translational phosphorylation of MabA by Ser/Thr protein kinase activity has been shown to negatively regulate mycolic acid biosynthesis [10]. The dehydratases of *M. tuberculosis* FAS-II (HadAB and HadBC) and methyltransferases have recently been shown to be part of a mycolic acid biosynthesis interactome, which is involved in coordinating elongation and chemical modification of mycolic acids [11]. For enzyme-targeted drug programs, however, mechanistic analysis should always be a top priority because effective enzyme inhibitors take advantage of enzyme chemistry to achieve inhibition [12]. As MabA represents a possible target for anti-tubercular agent development, the work here described was undertaken.

MabA belongs to the family of short-chain dehydrogenases/Reductase (SDR) [13], displaying a preference for NADPH over NADH, and higher specificity for long β-ketoacyl chains [14]. Crystal structure of MabA revealed a conserved Rossmann fold and the Ser-Tyr-Lys catalytic triad conserved among SDR members [15]. The conserved catalytic triad of MabA corresponds to the following signature: S140(X)_12_Y153(X)_3_ K157. The catalytic triads of SDR proteins catalyze abstraction of the proton from the substrate grouping H-C-O-H, although they also catalyze reduction of C = C and C = N double bonds, and mediate dehydratase, as well as sulfotransferase, isomerase, and decarboxylation reactions [16]. The dependence of MabA initial velocity on pH values has identified a single enzyme group with a p*K*_a_ value of 9.6 that abolishes activity upon deprotonation, which has been proposed to likely be either Tyr or Lys component of the Ser-Tyr-Lys triad [17]. However, it has been suggested that the Ser138 of the catalytic triad plays a catalytic role in *Drosophila* alcohol dehydrogenase, an SDR protein [18]. On the other hand, the Ser residues of the catalytic triad of SDR proteins have been suggested to play a minor role, if any, in catalysis [19]. Analysis of MabA crystal structure in the apo form showed a 90° rotation of Tyr153 phenol ring that was proposed to be induced by the hydroxyl group of “catalytic” Ser140 [15]. In this particular arrangement, S140 is placed into the position occupied by the nicotinamide ribose of NADP in the holo-form [15]. However, there has been no report on the role, if any, of S140 in the mode of binding and/or catalysis of MabA.

#### Side-directed mutagenesis, recombinant protein expression and purification

To evaluate the role of S140 residue in MabA, S140T and S140A mutants were produced by site-directed mutagenesis. The singly mutated genes corresponding to S140T and S140A were generated using the Quick Change Site-Directed Mutagenesis Kit (Stratagene) according to the manufacturer’s instructions and pET23a(+):*mabA* as the template. The mutant genes were sequenced in their entirety to ensure that no unexpected mutations occurred. The recombinant plasmid was transformed into *Escherichia coli* BL21(DE3) cells (Novagen) and grown in Luria-Bertani medium 50 g mL^-1^ carbenicillin, at 37°C to a value of 0.4 for absorbance at 600 nm, and induced by the addition of isopropyl-1-thio-*β*-D-galactopyranoside (IPTG) to a final concentration of 0.1 mM. Cells were allowed to grow for an additional 4 h and harvested by centrifugation at 20,800 *g* for 30 min. Soluble S140T and S140A mutant MabA proteins were purified to homogeneity as described elsewhere [17,20]. Samples of the purification steps were analyzed by SDS-PAGE [21] and protein content by the Bradford’s method [22].

#### Steady-state kinetics measurements

Activity assays of homogeneous S140T and S140A mutant enzymes were carried out under steady-state conditions at 25°C and 100 mM HEPES, pH 7.0, measuring decrease in absorbance at 370 nm (ε = 2,320 M^-1^ cm^-1^) upon oxidation of NADPH (1.5 – 6 mM) in the presence of acetoacetyl-CoA (AcAcCoA: 2 – 8 mM). These concentrations are well above the *K*_M_ values for NADPH (26 μM) and AcAcCoA (165 μM) [17]. In addition, increasing mutant enzyme concentrations (0.48 μM ≤ S140T ≤ 3.45 μM; 0.36 μM ≤ S140A ≤ 2.6 μM) were tested to try to detect β-ketoacyl Reductase activity, if any. The S140T and S140A mutants had no detectable activity under these experimental conditions.

#### Fluorescence spectroscopy

**Figure 1:**
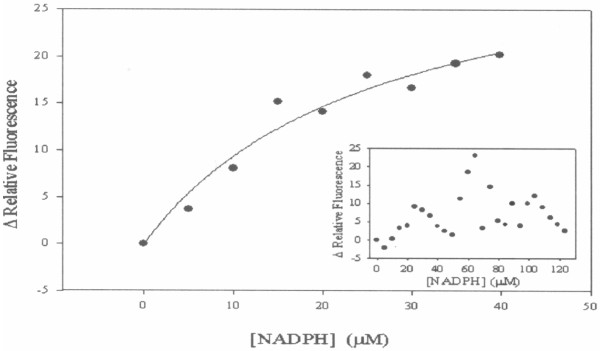
**Fluorescence spectroscopy of equilibrium binding of NADPH to MabA mutant proteins.** Dependence of the enhancement in nucleotide fluorescence upon NADPH binding to S140A mutant. Inset: NADPH titration of S140T mutant protein.

To ascertain whether these mutations affected catalysis or substrate binding, enhancement in nucleotide fluorescence upon NADPH binding to MabA was employed to evaluate the overall dissociation constant for binary complex formation at equilibrium (*K*_d_), as previously described [20]. Fluorescence titration of NADPH binding to either S140T or S140A mutants at equilibrium was carried out at 25°C by making micro liter additions of 10 mM NADPH to 2 mL of 4 μM mutant protein in 100 mM Hepes, pH 7.0, keeping the dilution to a maximum of 0.75%. Nucleotide fluorescence excitation and emission wavelengths were, respectively, 360 and 520 nm (maximum λ_em_ = 460 nm). The slits for excitation and emission were, respectively, 1.5 and 5 nm. Control measurements were performed under the same conditions, except that no enzyme was added, and these values were subtracted from those obtained in the presence of the enzyme. This control was also employed to determine the maximum NADPH concentration to be utilized with no significant inner filter effect. Data were plotted as Δ relative fluorescence versus NADPH concentration (4.99 – 123.45 μM for S140T, and 4.99 - 39.83 μM for S140A). Larger values for NADPH concentrations could not be used due to significant inner filter effect. No binding of NADPH to S140T mutant protein could be detected (Figure 1 - inset), indicating a *K*_d_ value larger than 123 μM. This result suggests that the S140T change resulted in impaired substrate binding and, consequently, catalysis. On the other hand, the S140A mutant protein could bind NADPH (Figure 1), and fitting the data to a hyperbolic function yielded an overall dissociation constant (*K*_d_) value of 26 (± 9) μM. This result suggests that S140 residue plays a role in catalysis. This result also demonstrates that the loss of enzyme activity of mutant protein is not due to gross structural changes.

#### Molecular modeling

Several attempts to obtain crystals for S140T and S140A MabA proteins were unsuccessful in our hands. Accordingly, to try to interpret the steady-state kinetics measurements and fluorescence spectroscopy results at a molecular level, bioinformatics tools were used. Homology modeling is based on the assumption that tertiary structures of two proteins will be similar if their sequences were related, and it is the approach most likely to give accurate results [23]. The homology models for S140T and S140A mutants in complex with NADPH were obtained using the coordinates of WT InhA as template (PDB ID 1UZN) and the restrained-based modeling implemented in the Modeler 9v5 program [24]. A total of 1000 models were generated and the final models were selected based on the objective function obtained from Modeler 9v5 [24]. The selected models were submitted to energy minimization in water, employing the GROMACS [25] package using Gromos 96.1 (53A6) force field [26] to make the analysis between protein and NADPH more realistic. Each structure was placed in the center of a truncated cubic box filled with Extended Simple Point Charge (SPC/E) water molecules [27], and the protein solvated by a layer of water molecules of at least 10 Å in all directions in both systems. The binary complex and water molecules were subjected to 1500 steps of energy minimization by conjugate gradient to remove close van der Waals contacts, and the overall stereochemical quality of the final models was assessed by the program PROCHECK [28]. Analysis of the Ramachandran diagram Φ-ψ plots indicated that all models had over 95% of the residues in the most favorable regions.

**Figure 2:**
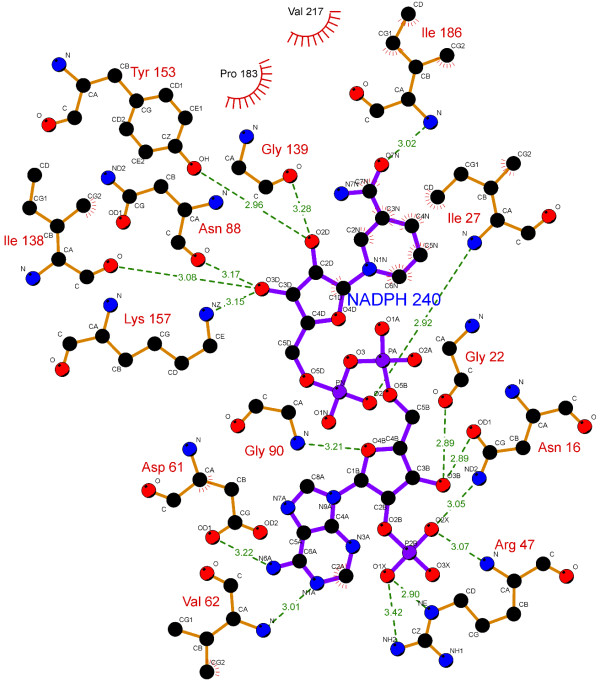
**LIGPLOT diagram for WT MabA and NADPH binary complex.** Covalent bonds of NADPH are in purple, covalent bonds of amino acids in light brown, hydrogen bonds are in green dashed lines. Protein residues in hydrophobic contacts with NADPH are represented by red semi-circles with radiating spokes. There are sixteen hydrogen bonds between WT MabA and NADPH.

**Figure 3:**
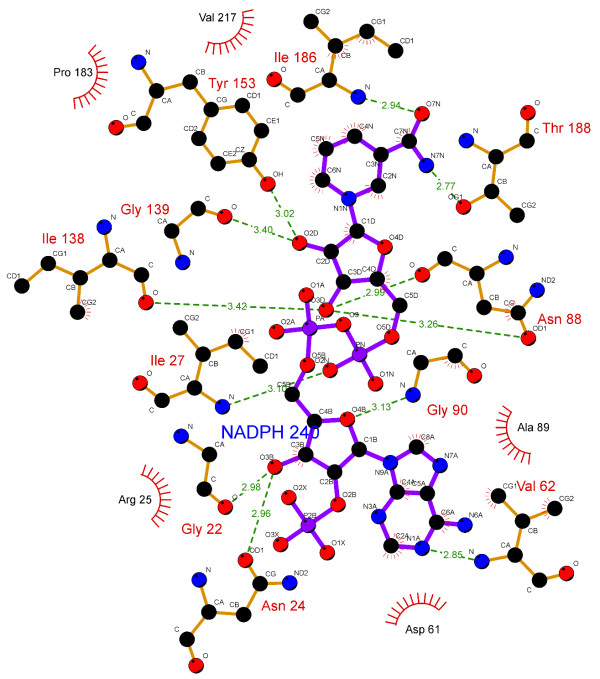
**LIGPLOT for S140T MabA mutant and NADPH binary complex.** Colour-coded bonds and intermolecular interactions are as for Figure 2. There are twelve hydrogen bonds between S140T MabA and NADPH.

**Figure 4:**
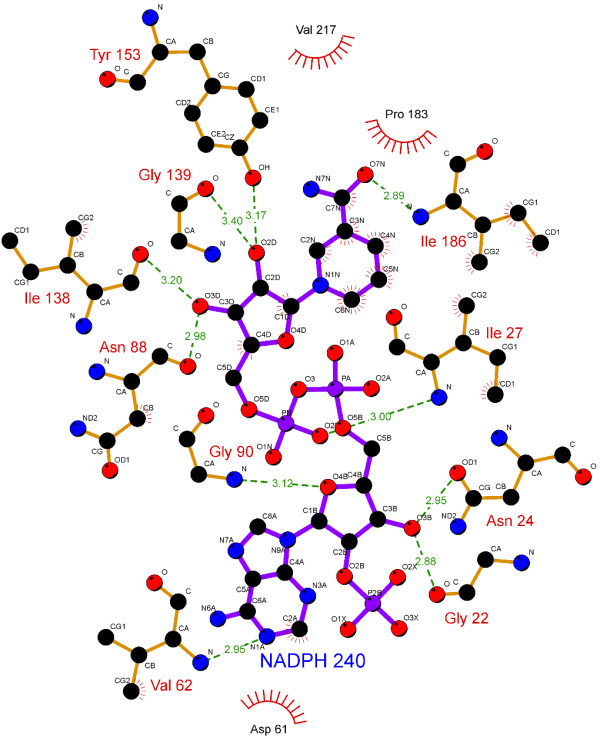
**LIGPLOT for S140A MabA mutant and NADPH binary complex.** Colour-coded bonds and intermolecular interactions are as for Figure 2. There are ten hydrogen bonds between S140A MabA and NADPH.

**Figure 5:**
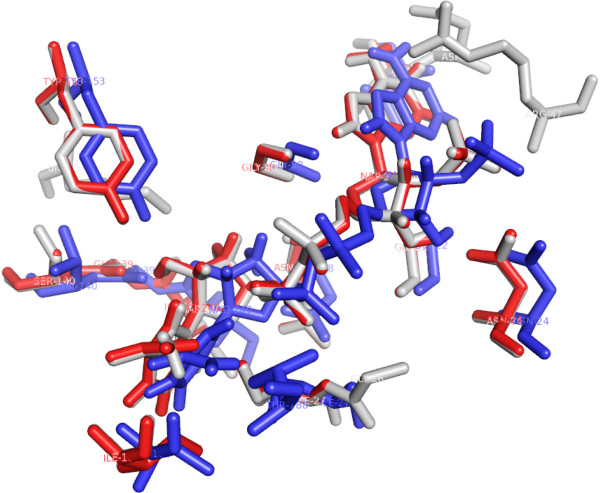
**The NADPH binding sites of WT MabA, S140A and S140T models are shown superimposed.** The structures are represented in stick model. WT MabA (light gray), S140A (red), and S140T (blue).

The specificity and affinity between enzyme and its substrate depend on directional hydrogen bonds and ionic interactions, as well as on shape complementarity of the contact surfaces of both partners [29,30]. Analysis of the hydrogen bonds between either WT, S140T, or S140A MabA protein and NADPH (Table 1) reveals, respectively, sixteen (Figure 2), twelve (Figure 3), and ten (Figure 4) intermolecular hydrogen bonds. The WT MabA, S140A and S140T models are shown superimposed in Figure 5. The hydrogen bonds were calculated by LIGPLOT program [31] with a 3.5 Å cutoff. The XSCORE [32], LigScore [33], and Drugstore [34] programs were employed to evaluate the binding affinity of NADPH for WT and mutant MabA proteins. The computational predictions for p*K*_d_ (-log*K*_d_) values for WT MabA, S140A and S140T mutants for NADPH binary complex formation are given in Table 2.

**Table 1:** Hydrogen bonds between amino acid residues and NADPH for WT, S140A and S140T MabA proteins

	**Hydrogen bond**			**Hydrophobic contact**
**Protein**	**Residues**	**Atoms**	**Distance (Å)**	**Residues**
MtMabAWT	Gly22	O → O3B	2.9	Pro183
	Asn24	OD1 → O3B	2.9	Val217
	Ile27	N → O2N	2.9	
		ND2 → O2X	3.0	
	Arg47	NH2 → O1X	3.4	
		NE → O1X	2.90	
		N → O2X	3.1	
	Asp61	OD1 → N6A	3.2	
	Val62	N → N1A	3.0	
	Gly90	N → O4B	3.2	
	Asn88	O → O3D	3.2	
	Ile138	O → O3D	3.1	
	Gly139	N → O2D	3.3	
	Tyr153	OH → O2D	3.0	
	Ile186	N → O7N	3.0	
	Lys157	NZ → O3D	3.1	
MtMabAS140A	Gly22	O → O3B	2.9	Asp61
	Asn24	OD1 → O3B	2.9	Pro183
	Ile27	N → O2N	3.0	Val217
	Val62	N → N1A	2.9	
	Asn88	O → O3D	3.0	
	Gly90	N → O4B	3.1	
	Ile138	O → O3D	3.2	
	Gly139	N → O2D	3.4	
	Tyr153	OH → O2D	3.2	
	Ile186	N → O7N	2.9	
MtMabAS140T	Gly22	O → O3B	3.0	Asp61
	Asn24	OD1 → O3B	3.0	Arg25
	Ile27	N → O2N	3.1	Ala89
	Val62	N → N1A	2.8	Pro183
	Asn88	O → O3D	3.0	Val217
		OD1 → O3	3.3	
	Gly90	N → O4B	3.1	
	Ile138	O → O3D	3.4	
	Gly139	O → O2D	3.4	
	Tyr153	OH → O2D	3.0	
	Ile186	N → O7N	2.9	
	Tyr188	OG1 → N7N	2.8	

**Table 2:** XSCORE, DrugScore and LigScore evaluation upon binary complex formation

	**XSCORE**^**a**^	**DrugScore**	**LigScore**
MabAWT	7.15	-139	-14,57
MabAS140A	7.06	-136	-12,18
MabAS140T	6.10	-119	-9,30

^a^ Given in units of pK_d_.

### Discussion

Although a component of the Ser-Tyr-Lys catalytic triad has been proposed to play a role in either catalysis and/or binding based on the dependence of WT MabA enzyme activity on various pH values [17], there has been no report on site-directed mutagenesis to unambiguously assign any role to the S140 residue of MabA. Pre-steady state kinetics of WT MabA: NADPH binary complex formation has shown that there are two forms of free WT MabA in equilibrium in solution with different affinity for NADPH [20], consistent with the symmetry model [35]. Equilibrium NADPH binding to WT MabA was shown to display strong positive homotropic cooperativity, with values of 47 μM for *K*', the mean dissociation constant for NADPH:WT MabA binary complex formation, and 2 for *h*, the Hill coefficient [20]. This Hill coefficient value is the upper limit for dimeric proteins, indicating complete positive cooperativity. An assumption of all-or-none (as in the symmetry model) is implicit in the Hill equation [36]. Accordingly, in the simplest case of strong positive homotropic cooperativity, an estimate for the intrinsic dissociation constant of NADPH binding to WT MabA (considering *K* = *K*_1_*K*_2_) would have a value of 6.86 μM. The predicted p*K*_d_ values are in good agreement with fluorescence spectroscopy data, showing that larger p*K*_d_ values correspond to lower dissociation constant values for binary complex formation. It should be pointed out that even though the p*K*_d_ values estimated by the XSCORE [32], LigScore [33] and Drugstore [34] programs are different, they all show the same trend in overall dissociation constants for binary complex formation (Table 2). In addition, p*K*_d_ values do not give a good prediction of the experimental *K*_d_ values.

There have been numerous reports showing that the central acid–base catalyst in SDRs is a Tyr group that donates or abstracts a proton to/from the substrate [16]. To function as a base, tyrosine must be ionized (tyrosinate). However, the normal p*K*_a_ of Tyr is 10.2, which would make it difficult to ionize in neutral solutions. In the active site of SDR proteins, the p*K*_a_ of Tyr is lowered by approximately four pH units by the positive electrostatic field created by a conserved Lys and the quaternary nitrogen of oxidized, charged cofactor nicotinamide. The Lys ε-amino group is also involved in nicotinamide ribose binding. However, pH-dependence of WT MabA kinetic parameters indicated that a single enzyme group with p*K*_a_ value of 9.6 abolishes activity upon deprotonation [17]. This p*K*_a_ value, as pointed out above, prompted the authors to suggest that either Tyr or Lys of MabA catalytic triad plays a role in catalysis and/or substrate binding. These results underscore the need to present experimental data to support a predicted role to any amino acid residue, since there appears to be no need to lower the p*K*_a_ value of catalytic Tyr side chain of MabA. A conserved active site Asn residue and its main chain carbonyl group interacts with a water molecule that is in H-bonding distance to the active site Lys, thereby establishing a proton relay system connecting bulk solvent to the active site Tyr residue in SDR proteins [37]. Site-directed mutagenesis of Ser138 residue of the Gram-negative bacterium *Comamonas testosteroni* 3β/17β-hydroxysteroid dehydrogenase (3β/17β-HSD) protein, an SDR family member, has shown that S138A mutation resulted in complete loss of activity, whereas S138T mutant had steady-state kinetic parameters comparable to WT 3β/17β-HSD enzyme [38]. Based on these results, it was proposed that the hydroxyl group of S138 side chain is essential for catalytic activity, playing a role in stabilization and/or polarization of carbonyl substrate and hydrogen bonding to the hydroxyl group of the conserved Tyr [38]. These authors have also pointed out that “it is not clear why in all SDR sequences thus far characterized Thr is conspicuously absent at this position, since our *in vitro* replacement of Ser138 with Thr yielded an active protein with identical catalytic constants”. However, here we show that the MabA S140T mutant has impaired NADPH binding and no enzyme activity could be detected, suggesting that, contrary to 3β/17β-HSD, Thr cannot replace Ser140 in MabA. It is tempting to suggest that impaired NADPH binding of S140T mutant as compared to WT and S140A MabA proteins is due to the bulkier side chain of threonine, which is borne out by the weaker intermolecular contact between NADPH and enzyme binding site (Figure 5). Although the S140A mutant is able to bind NADPH, this mutation results in inactive MabA enzyme. Accordingly, these results point to S140 residue of MabA as a residue whose main role appears to be in catalysis, in agreement with S138 of the catalytic triad of *Drosophila* alcohol dehydrogenase, also an SDR protein [18]. Notwithstanding, the S140 side chain may also play an indirect role in NADPH binding as the S140T showed impaired dinucleotide binding, in agreement with both the MabA: NADPH (Figures 2, 3, 4 and 5) and MabA:cofactor: substrate molecular models [39]. Interestingly, a conspicuous consequence of replacing S140 with threonine (Figure 3) or alanine (Figure 4) is on Lys157 which is no longer within the 3.5 Å cutoff for hydrogen bonding distance. Accordingly, the S140 residue appears to anchor the Lys157 catalytic triad of MabA. On the other hand, the S140 residue appears to have no effect on the Tyr153 of the catalytic triad of MabA. It has been proposed that a switch from “closed” to “open” conformation of MabA upon NADPH binding brings the phenol ring of catalytic Tyr153 side chain to the active site and that Ser140 induces this rearrangement [38]. However, we have recently shown that there are two forms of MabA in solution that differ in affinity for NADPH [20], which could imply that the “open” and “closed” conformations exist in equilibrium prior to NADPH binding. Interestingly, although tetrameric β-ketoacyl Reductase from *Escherichia coli*[40], *Plasmodium falciparum*[41] and *Brassica napus*[42] display negative homotropic cooperativity; β-ketoacyl Reductase from *M. tuberculosis* (MabA) [20] and *Staphylococcus aureus*[43] exhibit positive homotropic cooperativity. Unfortunately, attempts to obtain crystals of S140T and S140A MabA mutants have been unsuccessful in our hands.

### Conclusions

The S140 side chain appears to play a role in MabA catalysis that is likely not lowering the p*K*_a_ value of Tyr153. Interestingly, there was a shift from sigmoidal for WT MabA [20] to hyperbolic for S140A (Figure 1) titration curve upon NADPH binding. This finding suggests that the S140 residue may play a role in displacing the pre-existing equilibrium between two forms of MabA in solution, thereby affecting enzyme activity. Nina M. Goodey and Stephen J. Benkovic have pointed out that chemical compounds that can shift the equilibrium to protein conformers with lower substrate affinity and/or less catalytically competent must also be considered in drug design [44]. Ideally though, the crystal structure of S140A mutant protein should be obtained to shed further light on the role of this residue in MabA.

## Abbreviations

TB: Tuberculosis; MDR-TB: Multi-Drug Resistant TB; XDR-TB: Extensively Drug-Resistant; FAS: Fatty Acid biosynthesis System; MabA: *Mycobacterium tuberculosis*β-ketoacyl-ACP Reductase; SDR: Short-chain Dehydrogenases/Reductase; *K*_d_: Overall dissociation constant; 3β/17β-HSD: *Comamonas testosteroni* 3β/17β-hydroxysteroid dehydrogenase.

## Competing interests

The authors declare that they have no competing interests.

## Authors’ contributions

Conceived and designed the experiments: LAR, RAC, WFA, DSS and LAB. Performed the experiments: LAR and RAC. Analyzed the data: LAR, RAC and LAB. Contributed reagents/materials/analysis tools: WFA, LAB and DSS. Wrote the paper: LAR, LAB and DSS. All authors read and approved the final manuscript.
